# Upper lip bite test for prediction of difficult airway: A systematic review

**DOI:** 10.12669/pjms.344.15364

**Published:** 2018

**Authors:** Elnaz Faramarzi, Hassan Soleimanpour, Zahid Hussain Khan, Ata Mahmoodpoor, Sarvin Sanaie

**Affiliations:** 1 Elnaz Faramarzi Assistant Professor, Liver and Gastrointestinal Disease Research Center, Tabriz University of Medical Sciences, Tabriz, Iran; 2 Hassan Soleimanpour Professor of Anesthesiology, Department of Emergency Medicine, Faculty of Medicine, Tabriz University of Medical Sciences, Tabriz, Iran; 3 Zahid Hussain Khan Professor of Anesthesiology, Department of Anesthesiology, Faculty of Medicine, Tehran University of Medical Sciences, Tehran, Iran; 4 Ata Mahmoodpoor Associate Professor of Anesthesiology, Fellowship of Critical Care Medicine, Department of Anesthesiology, Faculty of Medicine, Tabriz University of Medical Sciences, Tabriz, Iran; 5 Sarvin Sanaie Assistant Professor, Tuberculosis and Lung Disease Research Center, Tabriz University of Medical Sciences, Tabriz, Iran

**Keywords:** upper lip bite test, upper lip catch test, prediction, difficult airway, difficult laryngoscopy, difficult intubation

## Abstract

**Background and Objective:**

Upper lip bite test (ULBT) is one of the various bedside tests used for prediction of difficult laryngoscopic intubation. However, its usefulness is not still very clear, and there is controversy regarding its accuracy. The aim of this systematic review was to determine the accuracy of the ULBT for predicting difficult airway including difficult laryngoscopy or difficult tracheal intubation.

**Method:**

We searched the databases of PubMed, Scopus, and Google scholar for prospective studies published up until October 2016 assessing the accuracy of ULBT in comparison to Cormack-Lehane grading. The selected keywords were “upper lip bite test”, “upper lip catch test”, “prediction”, “difficult airway”, “difficult laryngoscopy”, “difficult intubation”. Inclusion criteria were studies assessing ULBT for prediction of difficult intubation, considering Cormack-Lehane grade III and IV as difficult airway, written in English, and reporting sensitivity, specificity, NPV, PPV, and accuracy. Exclusion criteria were studies not reporting accuracy or not having enough data for its calculation. Based on the mentioned criteria, 27 studies enrolling 18141 patients were included. This systematic review was performed based on the guidelines on conducting systematic reviews of diagnostic studies.

**Results:**

Prevalence of airway difficulties according to the direct laryngoscopic view varied from 2.8% to 27% and according to the ULBT was from 2% to 21%. In 11 of the 27 studies, sensitivity of ULBT in prediction of difficult airway was more than 70%. All of the studies except one showed a high specificity for ULBT (>85%). Moreover, these studies indicated a high NPV. Accuracy of ULBT was >85% in 24 out of 27 studies.

**Conclusion:**

It appears that ULBT is a useful bedside test for evaluation of patient airway before the general anesthesia.

## INTRODUCTION

Difficult laryngoscopy and difficult tracheal intubation occur in 1.5% to 13% of patients undergoing general anesthesia and have always been a concern for anesthesiologists.^1^ Different method has been introduced by physician for management of difficult airway. However, the important note is the early and accurate detection of difficult airway for its safe management because failed intubation can have serious consequences and lead to high morbidity and mortality of the patients.^2^,^3^ Various bedside tests have been used for prediction of difficult laryngoscopy and intubation; of which, upper lip bite test (ULBT) has been proposed by Khan ZH et al as a good predictor for difficult laryngoscopic intubation.^4^ However, its usefulness is not still very clear, as various studies have demonstrated different results regarding its diagnostic accuracy. In a prospective blinded study comparing the ULBT with modified Mallampati test (MMT), ULBT significantly showed higher accuracy and specificity than MMT (*P* < 0.001). However, there were no significant differences in sensitivity, positive and negative predictive values between two tests (P>0.05).^4^ In another study, comparing ULBT with measurement of sternomental distance (SMD), thyromental distance (TMD), and interincisor distance (IID), it was revealed that the specificity and accuracy of the ULBT is significantly higher than the older tests. Also, ULBT, when combined with SMD, showed the highest sensitivity.^5^ A study evaluated the role of ULBT, MMT and TMD individually and also in various combinations in prediction of difficult laryngoscopy. Unlike the previous studies, this study showed that none of these three tests is a suitable predictive test when it is used alone. However, higher diagnostic value is achieved when they are combined together.^6^ Furthermore, the accuracy and reliability of the ULBT may vary according to patients’ sex and ethnic group; as lip size varies among different ethnicities. In addition, patients with collagen lip injections might show false positives or false negative results.^7^ The aim of this systematic review was to determine the accuracy of the ULBT for predicting difficult airway including difficult laryngoscopy or difficult tracheal intubation. The null hypothesis was that ULBT had poor accuracy for identifying difficult airway.

## METHOD

### Data sources

We searched the databases of PubMed, Scopus, and Google scholar for articles published up until October 2016. Key words were selected based on Mesh terms and included “upper lip bite test”, “upper lip catch test”, “prediction”, “difficult airway”, “difficult laryngoscopy”, “difficult intubation”. The manual search of the references of eligible articles for additional studies which were not identified by the electronic search was performed.

### Study selection

**Fig.1:**
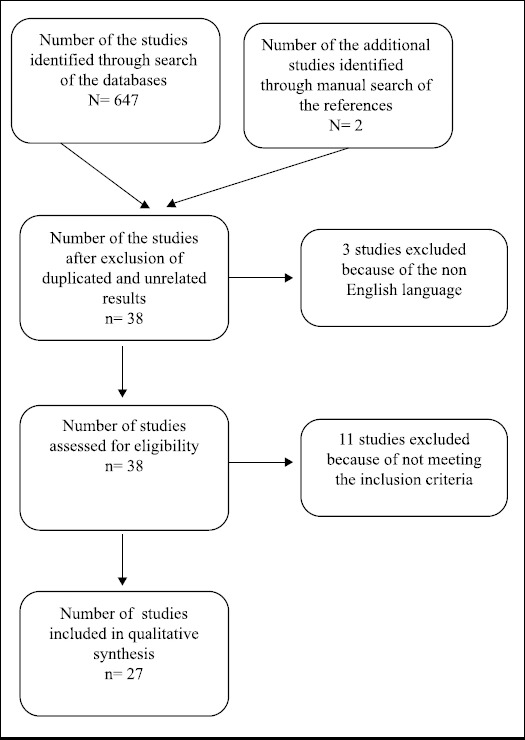
Flow diagram of the database search process.

Our inclusion criteria were as followings: prospective observational studies assessing preoperative ULBT to predict difficult intubation in patients undergoing general anesthesia, articles in English language, and studies reporting sensitivity, specificity, NPV, PPV, and accuracy. Albeit, some studies had not reported the accuracy; so, we calculated the accuracy based on the given results, where possible. If there were not enough data for its calculation, the study was not included to the present review. For all studies, Cormack-Lehane grade III and IV was considered as the gold standard. Difficult airway was defined by grade III score in the ULBT and the studies that reported grade II and III as the difficult airway was excluded from the study. The flow diagram of the study is presented in Fig.1.

### Data extraction

This systematic review was performed based on the guidelines on conducting systematic reviews of diagnostic studies.^8^

## RESULTS

The results of our search involved 27 studies (9-33) based on our inclusion criteria, as shown in Fig.1. Accuracy, sensitivity, specificity, PPV, and NPV of ULBT for each study are presented in Table-I. The total number of patients included in this systematic review is 18141 aging ≥15 years. Prevalence of airway difficulties in the reviewed studies according to Cormack-Lehane grading and also ULBT grading is presented in Table-I. In 11 of the 27 studies, sensitivity of ULBT in prediction of difficult airway compared to the gold standard was more than 70%. All of the studies except one showed a high specificity for ULBT (>85%). Moreover, these studies indicated a high NPV. Accuracy of ULBT was >85% in 24 out of 27 studies.

**Table-I:** Studies included in the systematic review.

Study (year)	Number of patients	Prevalence of airway difficulties according to reference standard (%)	Prevalence of airway difficulties according to ULBT (%)	Accuracy %	Sensitivity %	Specificity %	PPV%	NPV %
Khan et al. (2003)^4^	300	17 (5.7)	45(15)	88	76.5	88.7	28.9	98.4
Chohedri et al. (2005)^26^	500	14(2.8)	10(2)	96	14.2	98.3	20	97.5
Hester et al. (2007)^18^	50	9(18)	6(12)	90	55	97	83	90
Allahyary et al. (2008)^12^	203	37(18.2)	39(19.2)	97	94.6	97.6	89.7	98.8
Honarmand et al. (2008)^5^	400	35(8.75)	17(4.25)	90	17.1	96.9	35.3	92.2
Khan et al. (2009)^5^	380	19(5)	45(11.8)	91	78.9	91.9	33.3	98.8
Myneni et al. (2010)^20^	5999	173(2.88)	171(2.85)	94.73	8.1	97.6	8.2	97.6
Karnjanawanichkul et al. (2010)^27^	400	55(13.75)	9(2.25)	85.15	7.14	98.54	44.44	86.70
Sharma et al. (2010)^28^	62Acromegaly	15(24)	9(14)	74.2	26.7	89.4	44.4	79.2
	63 control	6(9)	12(19)	84.1	67	85.9	33	96.1
Khan et al. (2011)^21^	300	38(11.3)	16(5.3)	94	47.1	100	100	93.7
Ali et al. (2012)^13^	324	56(17.3)	68(21)	91.97	87.5	92.9	71.6	97.3
Khan et al.(2013)^14^	4500	265(5.88)	576(12.8)	90.91	81.5	91.4	37.5	98.7
Salimi et al. (2008)^29^	350	20(5.7)	36(10.3)	92.6	70	93.3	39	98.1
Shah et al. (2013)^22^	480	67(13.95)	85(17.7)	89.16	74.63	91.53	58.82	95.7
Mohan et al.(2013)^30^	140	15(10.71)	9(6.42)	91.42	40	97.6	66.7	93.12
Shah et al. (2014)^15^	450	47(10.4)	43(9.55)	95.5	91.5	96	72.8	98.9
Srinivasa et al. (2014)^31^	100	NS*	NS	91	77.14	98.46	96.43	88.89
Mehta et al. (2014)^23^	450	32(7)	16 (3.5)	95.1	50	98.56	72.77	92.26
Kolarkar et al. (2015)^16^	300	40(13.33)	18(6)	92.67	100	45	92.2	100
Honarmand et al. (2015)^24^	600	88(14.5)	46(8)	92	48.86	99.41	93.5	91.9
Javaherforoosh et al. (2015)^32^	448	38(8.4)	29(6.47)	89.95	28.9	95.6	37.9	93.5
Sharma et al. (2015)^25^	150	8(5.33)	4 (2.66)	94.66	25	98.6	50	95.9
Vallem et al. (2015)	200	54(27)	6(3)	73.5	5.66	97.9	50	74.14
Min et al. (2016)^33^	243	35(14.4)	18(7.4)	84.77	22.9	95.2	44.4	88
Aswar et al. (2016)^34^	200	16(8)	13(6.5)	89.5	25	95.11	30.77	93.58
Varghese et al. (2017)^35^	199	16(8)	5(2.5)	92	18.8	98.9	60	93.2
Sangeeta et al. (2016)^17^	350	30(8.57)	36(10.29)	94.85	80	96.25	66.66	98.08

* NS

## DISCUSSION

Incidence of a difficult laryngoscopy or endotracheal intubation is reported to vary from 1.5% to 13%.^1^ Difficult or failed intubation is a major cause of related anesthesia mortality.^1^-^3^ Therefore, airway management is a considerable challenge in anesthesia and preoperative airway assessment facilitates has a very important role in prediction of difficult laryngoscopy. There are many preoperative tests for prediction of difficult intubation. The most common are the Mallampati classification, TMD, SMD, IID and maximum mouth opening test;^9^,^10^ none of them being ideal compared to direct laryngoscopic view (Gold standard). Due to important roles of the range of freedom of the mandibular movement and the architecture of the teeth in facilitating laryngoscopic intubation, ULBT was introduced by Khan et al as a good predictor for difficult laryngoscopic intubation.^4^ Taking into account that an ideal test for prediction of difficult airway is the one with high sensitivity and specificity, few false positive predictions and of course, easy to perfume, different studies have evaluated the diagnostic value of ULBT. The results of these studies are inconsistent. Therefore, we evaluated the accuracy of ULBT for the prediction of difficult airway in this systematic review. The 27 included studies described 18141 patients in whom difficult airway is evaluated by ULBT. The reference test was Cormack-Lehane grading system in all of the studies^11^

Prevalence of airway difficulties according to the reference standard varied from 2.8% to 27%^5^ and according to the ULBT was from 2% to 21%. Significant variability in sensitivity and specificity was reported by the studies. However, ULBT had an overall high specificity and moderate level of sensitivity in these studies. In 11 out of 27 studies, sensitivity of ULBT in prediction of difficult airway compared to the gold standard was more than 70%.^4^,^5^,^12^-^17^ The moderate sensitivity of ULBT means that this test will not identify several patients who present with difficult intubation in Cormack-Lehane grading (smaller number of patients with true positive and larger numbers with false negative in ULBT). All studies except one of them showed high specificity for ULBT (>85%). Moreover, these studies indicated a high NPV. These findings is due to high true negative number; indicating high ability of this test to diagnose the patients who do not have difficult airway and therefore is a good test for detection of ease of laryngoscopy. Based on the formula used for accuracy calculation which involves true positive and true negative of patients with difficult airway, a test with high accuracy is an optimal test for prediction of difficult laryngoscopy. We observed a high accuracy of ULBT (>85%) in 24 of 27 studies meaning that ULBT has an optimal diagnostic value in preoperative assessment of patients candidate for general anesthesia.^4^,^5^,^12^-^14^,^17^-^25^

### Strength and limitation of the study

The strength of this study is that we reported the findings of studies that compared ULBT with Cormack-Lehane grading, not the ones comparing ULBT with other predictive tests. In addition, we evaluated the accuracy of ULBT used as a single test to achieve precise results; as ULBT has been assessed in combination with other tests in some studies. In these cases, it is not possible to attribute the results to ULBT alone. Because of heterogenicity of the studies, we were not able to conduct a meta-analysis on our findings which is the limitation of the present study.

## CONCLUSION

ULBT has moderate sensitivity and PPV, and high specificity, NPV and accuracy. So, it appears that ULBT is a useful bedside test for evaluation of patient airway before the general anesthesia. However, we suggest performing further studies with homogenous patients to achieve more clear results and to carry out a meta-analysis on the results.
